# Targeting the Hippo Pathway in Prostate Cancer: What’s New?

**DOI:** 10.3390/cancers13040611

**Published:** 2021-02-04

**Authors:** Kelly Coffey

**Affiliations:** Solid Tumour Target Discovery Laboratory, Translational and Clinical Research Institute, Newcastle University Centre for Cancer, Faculty of Medical Sciences, Newcastle University, Newcastle upon Tyne NE2 4HH, UK; kelly.coffey@newcastle.ac.uk

**Keywords:** Hippo pathway, prostate cancer, YAP/TAZ, cell signalling

## Abstract

**Simple Summary:**

Prostate cancer is the most commonly diagnosed cancer in men in the UK, accounting for the deaths of over 11,000 men per year. A major problem in this disease are tumours which no longer respond to available treatments. Understanding how this occurs will reveal new ways to treat these patients. In this review, the latest findings regarding a particular group of cellular factors which make up a signalling network called the Hippo pathway will be described. Accumulating evidence suggests that this network contributes to prostate cancer progression and resistance to current treatments. Identifying how this pathway can be targeted with drugs is a promising area of research to improve the treatment of prostate cancer.

**Abstract:**

Identifying novel therapeutic targets for the treatment of prostate cancer (PC) remains a key area of research. With the emergence of resistance to androgen receptor (AR)-targeting therapies, other signalling pathways which crosstalk with AR signalling are important. Over recent years, evidence has accumulated for targeting the Hippo signalling pathway. Discovered in *Drosophila melanogasta*, the Hippo pathway plays a role in the regulation of organ size, proliferation, migration and invasion. In response to a variety of stimuli, including cell–cell contact, nutrients and stress, a kinase cascade is activated, which includes STK4/3 and LATS1/2 to inhibit the effector proteins YAP and its paralogue TAZ. Transcription by their partner transcription factors is inhibited by modulation of YAP/TAZ cellular localisation and protein turnover. Trnascriptional enhanced associate domain (TEAD) transcription factors are their classical transcriptional partner but other transcription factors, including the AR, have been shown to be modulated by YAP/TAZ. In PC, this pathway can be dysregulated by a number of mechanisms, making it attractive for therapeutic intervention. This review looks at each component of the pathway with a focus on findings from the last year and discusses what knowledge can be applied to the field of PC.

## 1. Introduction

Prostate cancer (PC) is the most commonly diagnosed cancer in men in the UK, accounting for the deaths of over 11,000 men per year (Prostate Cancer UK). As an androgen-regulated cancer, initial treatments revolve around targeting the activity of the androgen receptor (AR) which is initially very effective. However, patients will become unresponsive to this treatment and go on to develop castration-resistant PC (CRPC). Treatments for PC have improved over the years with the introduction of second-generation anti-androgens such as enzalutamide; however, treatment relapse is still a problem. Therefore, there is still an unmet clinical need to develop novel treatments for CRPC and to understand the molecular pathways which lead to this disease status. In addition, with an increase in the occurrence of AR-negative neuroendocrine tumours (NEPC) following anti-androgen treatments, greater understanding of other cellular signalling pathways will yield novel therapeutic targets for these types of aggressive and fatal tumours.

**Figure 1:**
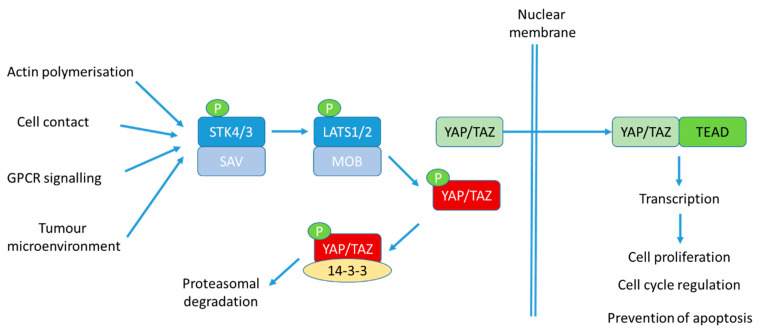
The Hippo pathway. In response to a variety of stimuli, upstream kinases STK4/3 are activated by their interaction with SAV to phosphorylate LATS1/2 and MOB1. LATS kinase activity is activated, resulting in phosphorylation of effector proteins YAP/TAZ. This results in cytoplasmic sequestration and interaction with the chaperone protein, 14-3-3, in the cytoplasm to inhibit its ability to promote transcription. Further phosphorylation of YAP/TAZ then occurs via CK1 to result in β-TrCP-mediated ubiquitination and proteasomal degradation. When YAP/TAZ cannot be phosphorylated, it resides in the nucleus where it can associate with transcription factors, such as Transcriptional Enhanced Associate Domain (TEAD) transcription factors, to stimulate transcription of genes involved in cell proliferation, cell cycle regulation, prevention of apoptosis, migration and invasion.

One such signalling pathway that has been gaining significant interest in PC research is the Hippo pathway (Figure 1). Indeed, this pathway is proving to be critical in many cancers. Originally discovered in the fruitfly, *Drosophila melanogaster* [1], its conservation across the species to regulate organ size, proliferation and stem cell biology makes its dysregulation an obvious interest to cancer biologists. The Hippo pathway is a cascade of kinase enzymes which, in response to various stimuli including cell–cell contact, nutrients and the surrounding microenvironment, inhibit downstream effector proteins, namely, YAP/TAZ, to switch off transcriptional programmes which promote cell growth. Critically, this is not the only method of regulation for these effector proteins which themselves have been found to be altered in cancers, including PC.

Recently, the Hippo pathway and its upstream activators were reviewed in great detail [2,3] and its role in PC was nicely reviewed by Salem and Hansen in early 2019 [4]. However, there has been a burst of activity in this field in the last year and this review will focus on providing a critical update in this fast-moving area of research, focusing primarily on PC and identifying where progress in other cancers could be applied to further advance our knowledge.

## 2. STK4/STK3

STK4 (MST1) and STK3 (MST2) are stress-activated kinases at the top of the kinase cascade which will ultimately regulate the activity of effector proteins, YAP/TAZ, as well as having Hippo-independent effects within the cell. STK4 is activated by autophosphorylation within its activation loop at Thr183 which in turn results in caspase-mediated cleavage under apoptotic conditions to produce a more activated form [5,6,7,8]. This form is then able to move to the nucleus of the cell where it results in activation of apoptotic gene expression via histone modification and chromatin condensation processes [9]. STK4 can directly interact with Akt1 to repress STK4 kinase activity [10] by phosphorylation at Thr120 resulting in inhibition of caspase-mediated cleavage [11]. With PTEN loss being observed in a large proportion of PCs, AKT activity is enhanced, suggesting a mechanism for SKT4 activity to be reduced in these cancers. Interestingly, STK4 has been shown to influence many oncogenic signalling pathways including AR signalling in PC [12].

**Figure 2:**
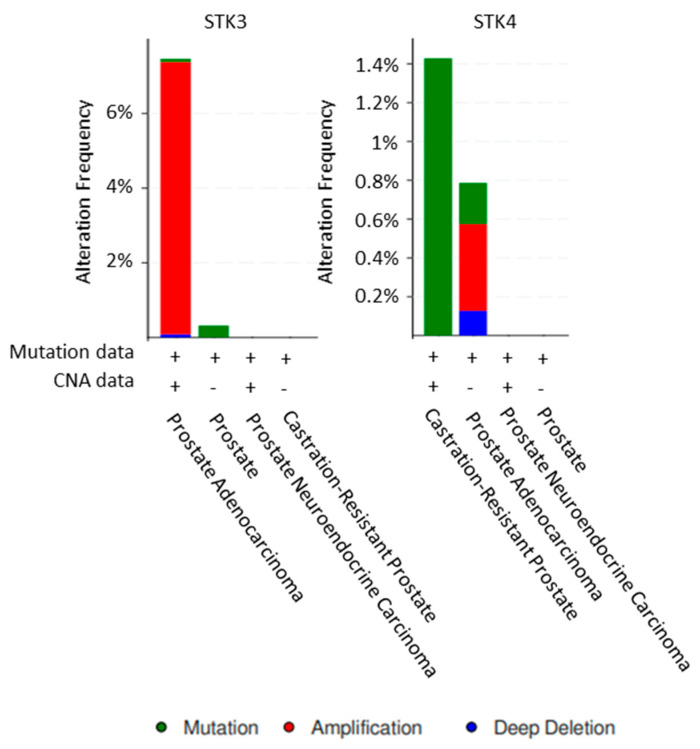
STK3 and STK4 alterations in prostate cancer. Analysis of all prostate cancer datasets within CBioportal [14].

STK4 has been found to be mutated in CRPC which results in decreased levels of LATS phosphorylation; however, only 11 patients were screened [13]. Yet STK4 contained mutations in around 1.4% of CRPC samples in CBioportal. The STK3 mutation rate is very low in prostate adenocarcinoma samples deposited in CBioportal, whilst 70 CRPC samples did not present any mutations. However, 7.3% of prostate adenocarcinomas had amplification of STK3 [14] (Figure 2).

MicroRNAs can play a role in the post-transcriptional regulation of STK4, in particular miR-18a, which is overexpressed in PC [15]. Interestingly, STK4 and STK3 are not detected at the protein level in prostate tissues in the Human Protein Atlas (www.proteinatlas.org) [16], and tissue microarray analysis performed by Cinar et al. showed a reduction in expression of STK4 in metastatic PC samples [10]. Low expression of these upstream kinases is thought to be due to epigenetic silencing as a consequence of c-Myc and EZH2 activity. Indeed, re-expression has been observed in response to stimuli such as JQ1, which down-regulates c-Myc [17]. Furthermore, protein turnover plays a role in the low protein expression of STK4. The heat shock protein, HSP27, can promote proteasomal degradation of STK4 which results in the failure of the kinase cascade to be activated. Moreover, STK4 down-regulation by HSP27 has been shown to mediate cisplatin resistance in PC cells [18]. HSP27 is up-regulated in PC and in particular in response to androgen ablation treatment (reviewed in [19]). As patients with high levels of HSP27 have a poor prognosis, this may well be associated with an impact on STK4 levels, which in turn prevents or reduces the ability of LATS to prevent YAP activation. Further supporting this theory, HSP27 has been shown to regulate the transcriptional programmes of YAP/TAZ and HSP27 loss does lead to a rise in STK4 protein levels in PC cells [20]. Therefore, regulation at the protein level appears to be important for STK4.

STK4 is a key mediator of signalling from Par3/Merlin/LATS2. Par3 (polarity protein) is a scaffold protein which plays a key role in the regulation of cell polarity, an upstream signal regulator of the Hippo pathway. In order for STK4 to be able to associate with LATS and permit phosphorylation and activation, Par3 is required. Par3 loss in combination with a signalling block of the Hippo pathway can result in initiation of PC [21]. Recently, IKBKE was shown to play a role in Par3 cellular localisation [22]. As IKBKE plays a role in the regulation of LATS turnover [23,24] as discussed below, further study of Par3/LATS/IKBKE may be warranted in PC.

## 3. LATS1/2 Kinases

LATS1/2 are tumour suppressor genes which have been well studied for their role in the Hippo pathway. Upstream kinases target a hydrophobic motif within the molecule which, upon phosphorylation, facilitates autophosphorylation of the activation loop. However, not all LATS phosphorylation events result in kinase activation; SRC can inhibit LATS via GTPase activating protein (GIT) [25] and via direct Tyr phosphorylation [26]. Regulation of LATS is primarily by post-translational mechanisms, but some transcriptional mechanisms have also been determined.

LATS expression can be regulated as part of a feedback loop from the Hippo effector proteins YAP/TAZ/TEAD and a role for miRNAs in LATS regulation has been discovered. Specifically, MiR302 and MiR367 have been shown to play a role in CRPC via LATS regulation which subsequently impacts the effector protein YAP [27]. Similarly, MiR-93 was discovered to regulate LATS in hepatocellular carcinoma and breast cancer. Upon investigation in PC, MiR-93 was found to be overexpressed [28]. Most recently, miR-15b-5b has also been shown to modulate LATS [29]. All studies demonstrate that these miRs are overexpressed in PC and result in a down-regulation in LATS to cause increased cellular proliferation, colony forming and invasion which can be rescued by overexpression of LATS2.

Reduced LATS expression is seen in tumours with FOXP3 mutations [30]. FOXP3 inhibits expression of ErbB2 [31], cMyc [32] and skp2 [33] and induces p21 [34] to inhibit cell growth. As cMyc, skp2 and LATS are all YAP-regulated genes, it would suggest that a FOXP3-mediated reduction in LATS down-regulates YAP activity. Loss of LATS1/2 in ovarian granulosa cells causes the ovarian parenchyma to be replaced with bone tissue and seminiferous tubule-like structures. Up-regulation of YAP/TAZ and their recruitment to sex-determining region Y box9 and bone gamma carboxyglutamate protein under these conditions result in transdifferentiation into Sertoli-like cells and osteoblasts [35]. This may suggest a role for loss of LATS1/2 in PC bone metastases.

### 3.1. Post-Translational Activation of LATS1/2

Activation of LATS activity is an important function in limiting cell proliferation stimulated by its effector proteins including YAP/TAZ. There is increasing evidence that activation of the Hippo pathway integrates with many cellular pathways, many of which are defective in cancers. Many of these pathways involve post-translational modification of LATS. Classically, phosphorylation by kinases such as STK4/3, TAOK kinases and MAP4K kinases has been shown to activate LATS kinase activity. Indeed, redundancy between these kinases is thought to be present and their individual roles dependent on cell type. Signals which initiate this cascade of phosphorylation events include cell–cell interaction, cellular stress and interaction with the extracellular matrix. Activation of LATS1/2 in non-transformed RWPE prostate cells by cell detachment resulted in down-regulation of YAP/TAZ activities to activate anoikis. This study determined that the activating event occurred downstream of STK3/4 but upstream of LATS1/2, but ruled out Src, FAK and Rho kinase, yet microtubule integrity was important [36]. Therefore, abnormal Hippo signalling may play a role in preventing anoikis and therefore promote metastatic capability. Due to the reported down-regulation of LATS1/2 in advanced PC, this could be a significant contributing factor.

Protein Tyrosine Phosphatase, non-receptor type 14 (PTPN14), which is regulated by cell–cell adhesion and cell–matrix adhesion, activates LATS1 to down-regulate YAP, resulting in inhibition of cellular proliferation. In PC, PTPN14 is down-regulated and upon re-expression, PTPN14 inhibits cellular proliferation via LATS1-mediated inhibition of YAP [37]. Interestingly, the deubiquitinase BAP1 has been shown to enhance the protein stability of LATS. Somatic mutations in BAP1 are rare in PC, similar to breast, gastric and colorectal cancers [38]; however, cellular localisation may be important, with nuclear BAP1 being associated with tumour aggressiveness in a subset of patients [39,40,41]. Contrary to this, Deng et al. recently published some similar experimentations with the opposite outcomes and demonstrated that BAP1 knockdown promotes tumourigenesis by stabilising PTEN [42]. This highlights that further investigations are needed and considerations regarding other signalling molecules may be important cofactors in what conclusions are drawn.

STK25 phosphorylates LATS within the activation loop. However, this is different to other kinases as phosphorylation within the hydrophobic motif is not involved in the activation process [43]. More recently, STK25 has been found to phosphorylate SAV1, resulting in increased PP2A activity to inhibit MST1 [44] and contradicting the findings of Lim et al. Therefore, further investigation is required to unravel the role of STK25 in these events. Loss of STK25, which is reported in many cancers, results in activation of YAP [43]. Yet upon investigation of STK25 deletion in CBioportal [14], a deep deletion frequency of 1% is reported and the only protein staining data in prostate tissue suggest an increase in STK25 is seen in cancer [45] which is contradictory to samples detailed in the Human Protein Atlas, where 7 samples out of 11 PCs had no detectable expression of STK25 (www.proteinatlas.org) [16]. However, mRNA expression is significantly up-regulated 3-fold in prostate carcinoma compared to a normal prostate gland in the Tomlins Prostate dataset [46] within Oncomine™. This may suggest a disconnect between STK25 mRNA levels and protein levels.

### 3.2. Post-Translation Repression of LATS1/2

Repression of LATS activity can also be achieved by post-translational modification. LATS2 levels inversely correlate with cancer stage in a number of malignancies including PC. The most important method of repressing LATS activity is via proteasomal degradation. Indeed, in the Human Protein Atlas, 9 out of 12 PC specimens did not contain any LATS2 protein (www.proteinatlas.org) [16]. Gα13-mediated phosphorylation of LATS at Serine 909 results in recruitment of the E3 ligase Itchy to result in LATS degradation in ovarian cancer cells [47]. Interestingly, ITCH is reported as down-regulated in PC and its loss is associated with tumour stage [48]. Yet conflicting data suggest ITCH promotes PC progression [49]. Therefore, it is impossible to speculate at this stage whether ITCH assists in the down-regulation of LATS in PC. Similarly, IKBKE can phosphorylate LATS to promote its turnover by the proteasome [24], an observation which was supported by IKBKE knockdown in PC cells whereby LATS expression was increased at the protein level [23]. Furthermore, with IKBKE levels elevated in cancers, including PC, aberrant IKBKE activity offers another explanation as to why LATS protein levels are down-regulated in cancers.

LATS activity can also be reduced by post-translational modifications which prevent interaction with its activating proteins. The PP2A regulatory subunit, PR55, inhibits MOB1 autoactivation of LATS1/2 to result in YAP activation in pancreatic cancer cells [50]. More recently, FAK has been shown to phosphorylate MOB1 at Y26, which causes it to dissociate from LATS to result in YAP activation [51]. Furthermore, modification at Thr436 with O-GlcNAc prevents LATS from interacting with MOB1-MST. This is observed in the presence of high glucose in breast cancer cells to prevent phosphorylation at Ser872 and Thr1041 which is critical for LATS kinase activation [52].

ALK has been discovered as a novel LATS inhibitor kinase using an inhibitor library screen. Further validation demonstrated that ALK inhibitors increased LATS activity and that overexpression of ALK suppressed activity [53]. ALK amplification is associated with poor outcome in PC and a clinically relevant mutation, F1174C, yielded enhanced growth and neurone-specific enolase (NSE) expression which may indicate a role in the development of NEPC [54]. Other kinases that were identified in this inhibitor screen included eEf2K, IRE1, SMG1, PIM and TrkA as positive regulators and TBK1, gamma-secretase, JAK2 and PLK as negative regulators [53].

GPR4, a G-protein coupled receptor which detects protons, is overexpressed in colorectal cancer and correlates with late-stage tumours and reduced overall survival. Upon detecting protons, GPR4 activates RhoA and results in F-actin rearrangement, which inhibits LATS activity [55]. When GPR4 is overexpressed in TRAMP-C1, cellular migration is inhibited [56] which may suggest a similar pathway exists in PC. RhoA activation can also occur in response to mechanical stresses to yield inhibition of LATS activity via the same pathway [57]. Heat stress is another extracellular stimuli that can result in LATS activation. Specifically, LATS is inactivated by PP5 in an HSP90-dependent manner. Interestingly, downstream of LATS, YAP can still be phosphorylated and inactivated by other kinases such as NLK and AMPK in response to their activating stimuli—energy starvation and osmotic stress, respectively [58]. Therefore, other kinases may also be able to influence YAP activity in the absence of LATS.

POPX2, a member of the PP2C family of Ser/Thr phosphatases, dephosphorylates Thr1079 to inactivate LATS kinase activity. The gene encoding POPX2, PPM1F, has been described as an annexin A1-regulated gene in PC. With an inversely related expression profile to the AR, it is suggested that annexin A1 may facilitate invasion and metastasis in response to androgen deprivation therapy [59]. Indeed, upon investigation of the Tomlins Prostate dataset [46] within Oncomine™, there is an up-regulation of POPX2 in hormone-refractory samples compared to hormone-naïve samples. Furthermore, in breast cancer cells, knockout of POPX2 impaired anchorage-independent growth [60]. Inactivation of LATS by dephosphorylation in pancreatic cancer is caused by PKCiota cooperation with mutant KRas [61], both of which are up-regulated in PC and associated with disease progression [62].

Another mechanism of LATS kinase activity regulation is by interaction with other proteins. CDH1, which is up-regulated in malignant tumours, associates with LATS and has been hypothesised to reduce its kinase activity due to the site of interaction. However, this has not been confirmed experimentally. Reduction in CDH1 does result in enhanced YAP phosphorylation and a corresponding down-regulation in the levels of YAP-regulated genes which supports this theory [63].

LATS can also directly phosphorylate other cellular components to influence other signalling pathways. For example, phosphorylation of Raptor at Ser606 results in attenuation of mTORC1 kinase activity. The consequence of this is inhibition of glycolysis and lipid biometabolism [64]. Interestingly, mTORC1 is a therapeutic target in PC and a recent study demonstrated that DEPTOR, whose phosphorylation by LATS promotes its inhibitory association with mTORC1, is reduced in PC at both the protein and mRNA levels [65].

## 4. YAP1

Downstream effectors of the Hippo pathway are classically YAP and its paralogue TAZ (WW domain containing transcription regulator protein 1). YAP functions as a transcription factor co-activator to modulate gene programmes that promote proliferation, migration and invasion. It is most studied alongside the TEAD family of transcription factors but can also associate with other transcription factors including the AR [66]. YAP activity is regulated by LATS-mediated phosphorylation to result in export from the nucleus where it binds to 14-3-3 mediated by S127 phosphorylation. LATS-mediated phosphorylation at Ser381 mediates phosphorylation by Casein Kinase (CK) to recruit the β-TrCP ubiquitin ligase, leading to destruction by the proteasome [67]. Similarly, TAZ is phosphorylated to undergo the same signalling process to inhibit its activity. In addition, NDR1/2 can phosphorylate YAP and the NDR1/2-associated protein FRY (furry) can associate with YAP to support cytosolic sequestration [68].

YAP is often overexpressed and hyperactivated in many cancers including PC; however, mechanisms which lead to hyperactivation were poorly understood. Recently, new mechanisms and hypotheses have been proposed, suggesting new targets for therapeutic intervention and/or patient stratification. Importantly, transcriptional regulation of YAP has been implicated in PC. In ~45% of PC patients, the presence of the gene fusion TMPRSS2-ERG results in the overexpression of ERG which is a transcription factor for YAP. In cell line models, up-regulation of ERG causes YAP promoter activation; however, this does not appear to be the case for mRNA in clinical samples where the presence of this gene fusion does not associate with increased YAP expression (www.oncomine.org) [69,70,71]. However, positive correlations between TMPRSS2-ERG fusions and YAP1 protein expression were observed in a large study of patient samples by immunohistochemistry [72]. Additionally, several miRNAs that are down-regulated in cancer have been discovered to target YAP including MiR-205, MiR-132 and MiR-16-1 [73]. More recently, a decrease in the YAP-targeting MiR-27a-15a-16, as a consequence of increased ZNFX1 anti-sense RNA 1 (ZFAS1) in PC, was discovered to enhance YAP levels to promote proliferation, invasion and EMT [74].

Enhanced protein stability appears to be a major mechanism by which YAP activity can be increased in cancers. MK5, also known as MAPKAPK5, has recently been shown to be a positive regulator of YAP in both mesothelioma and uveal melanoma, by its association with YAP preventing CK1δ/ε-mediated proteasomal degradation. In support of MK5′s oncogenic role, enhanced MK5 levels correlate with YAP levels, resulting in a poor prognosis [75]. However, independent proteomic analysis of YAP1 in the presence of MK5 revealed no novel phosphorylation sites, suggesting MK5 may not directly phosphorylate YAP [76]. Interestingly, MK5 is up-regulated in cancer versus normal samples within the Grasso Prostate dataset [71], suggesting further investigation may be warranted in PC. In contrast, NEK1 was found to phosphorylate YAP at multiple sites to result in enhanced protein stability [76]. Similarly, PIN1, which is overexpressed in PC [77], has been shown to enhance the stability of YAP/TAZ in breast cancer to produce taxol resistance [78]. Furthermore, in melanoma studies, PIN1 can interact with STK3 to induce its proteasomal turnover and plays an important role in TAZ nuclear localisation and association with TEAD to enhance TAZ-regulated gene expression [79].

Four studies have been published in 2020 detailing immunohisotchemical data for YAP/TAZ. The first used 203 tissue samples taken from 70 high-risk localised PCs, revealing that patients who underwent chemohormonal therapy (neoadjuvant chemohormonal therapy combined with androgen deprivation therapy, docetaxel and estramustine phosphate) had the highest levels of nuclear YAP. As expected, these patients also had the lowest level of nuclear AR staining; however, nuclear GR, cytoplasmic MOB4A and stromal PR were up-regulated. Interestingly, in residual cancer found after therapy where YAP levels were elevated, higher levels of biochemical recurrence were noted. A much larger study looking at 17,000 samples was performed by Marx et al. (2020) and revealed that high levels of both nuclear and cytoplasmic YAP1 associated with early biochemical relapse and advanced tumour stage, Gleason grade, positive surgical margins, increased Ki67 staining and positive nodal stage. Associations between YAP1 staining and increased AR expression, PTEN loss and 8p deletions were also discovered [72]. A smaller study compared the expression of YAP1 in 22 benign prostatic hyperplasia samples to increasing grades of prostate adenocarcinoma and 12 neuroendocrine biopsy samples. They found that YAP1 is localised to basal epithelial cells in normal prostate and its protein expression increased with grade in prostate adenocarcinoma. However, upon investigation of mRNA levels in NEPCs, expression of YAP1 appears to be reduced, whilst in 12 samples that were investigated for protein expression, 6 did not show expression, with the other 6 showing staining in less than 25% of cells [80]. Therefore, it would be useful to assess not only more samples to clarify any associations of YAP expression with the NEPC phenotype, but to also investigate both the protein and the transcript in the same samples to determine whether there is a discord between the two, particularly when protein stability appears to be a key regulating factor for YAP.

YAP monoubiquitination has been uncovered as another method by which subcellular localisation, and therefore activity, can be modulated. This occurs independent of Hippo signalling as the mutant YAP protein, which cannot be phosphorylated at S127 and therefore resides in the nucleus, can still be sequestered to the cytoplasm when sites of ubiquitination are also mutated [81]. Similarly, Mastermind-like (MAML), a coactivator of Notch-dependent transcription, can promote YAP/TAZ nuclear localisation at low cell density independent of Hippo signalling [82]. Investigation into whether MAML and monoubiquitination of YAP function in the same pathway has not been investigated. Monoubiquitination of YAP is performed by the E3 ubiquitin ligase complex SCF^Skp2^, which adds K63-linked ubiquitin to result in enhanced YAP activity by promoting YAP–TEAD interaction in the nucleus. Conversely, OTUD1 has been identified as the deubiquitinase which can remove this modification and return YAP to the cytoplasmic compartment [81]. Interestingly, S-phase kinase associated protein 2 (Skp2) has been identified as a YAP-regulated gene which suggests a positive feedback loop is present between YAP and skp2 [83]. Indeed, Skp2 is up-regulated in PC [84] and is involved in the development of drug resistance in cancer [85,86,87,88,89].

Other post-translational modifications of YAP include acetylation by p300/CBP at a number of lysine residues [90] and O-GlcNAcylation by O-GlcNAc transferase at Ser109 [91]. Acetylation impairs activation of YAP, whilst deacetylation by SIRT1 results in nuclear accumulation and enhances the association with TEAD4 in response to cisplatin treatment in HepG2 cells [90]. O-GlcNAcylation of YAP by O-GlcNAc transferase at Ser109 enhances YAP activity by preventing an interaction with the upstream kinase LATS in response to glucose [91]. Similar observations were made in liver cancer cells, where O-GlcNAcylation at Thr241 was observed [92].

FERM Domain Containing 6 (FRMD6), a protein involved in maintaining epithelial cell structure, can activate Hippo kinases to down-regulate YAP activity [93]. FRMD6 has recently been identified as a tumour suppressor gene in PC which is down-regulated in PC with low expression being associated with biochemical recurrence [94]. Co-expression of FRMD6 with ILK is highly correlated in the Taylor Prostate 3 dataset (0.809) [95]. ILK kinase activity can result in inactivation of NF2/Merlin and inhibition in prostate cells leads to activation of Hippo kinases to inactivate YAP/TAZ [96]. A connection between ILK/FRMD6 and YAP activity may require further investigation.

## 5. TAZ/WWTR1

The paralogue of YAP, TAZ, shares 46% homology with YAP. In a number studies, both YAP and TAZ are referred to as a pair as many antibodies detect both proteins due to their high similarity. Importantly, YAP and TAZ cannot compensate for each other [97]. Studies using a constitutively active TAZ found increased migration and colony forming ability but no enhancement of proliferation. This was accompanied by increased expression of E-Cadherin, FN1, vimentin and B-catenin [98]. TAZ can function independently of YAP to enhance DNA synthesis and also appears to be important in cell cycle regulation [99]. In lung cancer cells, MEKK5 has been found to prevent TAZ translocation to the nucleus, thereby preventing proliferation and migration independent of YAP [100]. TAZ activity can also be stimulated by shear stress which is increased in cells circulating in the blood stream [99], suggesting a role in the metastatic process. Hypoxia is reported to enhance phosphorylation of TAZ at Ser69 to down-regulate its activity, but phosphorylation of YAP is reduced, enhancing its activity in a number of cell lines including PC3 cells [101]. As hypoxia is associated with radioresistance in many cancers, it is interesting to note that in oesophageal cancer, TAZ regulates the expression of genes involved in non-homologous end joining that are a causative factor in radioresistance [102]. In addition, TAZ plays a role in EMT, migration and anchorage-independent growth in a number of cancers but it remains understudied in PC. The question of PC stage-specific expression has been answered in part by a study showing that both protein and transcript expressions associate with PC grade [103]. However, the loss of TAZ expression has been reported in PC and it has been proposed that when TAZ is re-expressed, it results in a more aggressive disease. Contrary to this, expression in NEPC is reported as reduced or lost [80]; therefore, further investigation is still required. In breast cancers, the deubiquitinase USP1 can interact with TAZ to cause increased protein stability [104]. USP1 is also proposed as a therapeutic target in prostate cancer [105], although its role in YAP/TAZ signalling has not been investigated.

## 6. Extracellular Matrix Sensing

The extracellular matrix within which cancer cells grow can influence the signalling pathways to influence cell growth, invasion and migration capabilities. The Hippo pathway has been shown in numerous studies to play a role in environment sensing. This can be particularly important during metastatic processes and may explain, in part, why PCs favour deposition in bone once they have escaped the primary tumour site. Cell growth on materials of different stiffness has revealed some interesting observations. A comparison of PC3 cells grown in tissue culture dishes to mimic a hard growth surface and on decellularised spinach leaves to mimic a soft growth surface revealed slowed proliferation, down-regulated YAP/TAZ signalling and also altered cellular morphology. Furthermore, the response to cellular stress, including radiation exposure, was altered, yet DNA repair was effective on both growth surfaces, suggesting that YAP can promote radioresistance in PC [106]. However, the origin of the cell can affect how a response is made. For example, cells derived from bone metastases show higher proliferation and migration on high-stiffness substrates, whereas lymphatic-derived cells grown on low-stiffness substrates see higher levels of proliferation and migration [107]. These findings highlight the importance of studying this pathway in vitro under the correct conditions to mimic where cells grow in the body.

## 7. Self-Renewal

Cancer stem cells are important in driving therapy resistance and transcription factors associated with pluripotency, specifically Nanog, Oct4 and Sox2, have been associated with aggressive PC [108]. In other cancers, deletion of LATS and the resultant up-regulation in YAP/TAZ activity resulted in uncontrolled expansion of Sox2-positive cells [109], whilst LATS1/2 activity can trigger self-renewal of cancer stem cells in aggressive oral cancer [110]. Therefore, it would be interesting to investigate the levels of Hippo pathway activity in this subgroup of patients with high expression of Oct 4, Sox2 and Nanog. TAZ is thought to be more important for stemness [111] and is found to be expressed at higher levels in PC3-derived cancer stem cells alongside phosphodiesterase 5 (PDE5). Indeed, both Sox2 and Nanog expressions were reduced in response to TAZ knockdown [112]. It is thought that PDE5 promotes TAZ activity as inhibition functions through PKG to activate STK4 to activate the Hippo pathway and result in TAZ inactivation [112]. This is contradicted by development studies which showed that transcription of Sox2 can be inhibited by YAP/TAZ and TEAD4 to prevent premature expression of Sox2, thereby limiting the pleuripotency gene programmes until day 16 [113]. This raises the question of whether a developmental process has been initiated in these PC3-derived stem cells which is malfunctioning so that instead of TAZ inhibiting Sox2, it is promoting its expression.

In intestinal stem cells, activation of TAZ by LATS deletion inhibits Wnt by interacting with Groucho/TLE to block Wnt/TCF-mediated transcription [114]. Conversely, YAP/TAZ have been shown to be under the control of the Wnt pathway and be downstream effectors [115,116]. As LATS and TAZ down-regulation is prevalent in PCs, this may partially explain why Wnt signalling is able to stimulate AR and YAP translocation to the nucleus under androgen deprivation conditions [117].

## 8. Cell Cycle

Oscillations in both LATS and YAP/TAZ activities are seen during the cell cycle, suggesting a role for the Hippo pathway in cell cycle regulation. Indeed, a delay in the progression through G1 to S is observed when YAP/TAZ is knocked down. LATS1/2 activity peaks during G2/M, whilst YAP/TAZ activity is highest during G1. During the G1 and S phases, TAZ protein levels are increased, whilst phosphor-YAP is decreased to result in higher nuclear localisation in comparison to G2/M phases [63]. This is particularly interesting as AR has been described as a driver of G1/S transition via transcriptional regulation [118].

## 9. Interplay with the AR

Interestingly, LATS2 has been shown to interact with the AR, whereby it can impede the interaction of the N- and C-terminal domains, which is important for transcriptional activation. Surprisingly, LATS2 interaction with AR appeared to occur in the presence of androgen stimulation which might suggest that activation of LATS2 kinase activity by stimuli such as cell–cell contact may signal to turn off AR activity. However, LATS2 kinase activity was not investigated in this process [119]. LATS can also associate with MDM2 which results in inhibition of its E3 ligase activity and activation of p53. MDM2 can also associate with AR to play a role in its ubiquitination and destruction by the proteasome. Whether LATS and MDM2 can work together in terms of AR signalling remains unstudied. As LATS interacts with AR in the presence of androgens and MDM2 can function to signal AR turnover, it would be interesting to test whether these two proteins work to turn off AR transcription at the chromatin level and play a role in AR cycling on and off chromatin.

The Hippo signalling pathway as a whole can modulate the activity of the AR. Not only can LATS, whose expression is often lost in PC, modulate AR activity but down-stream effectors can modulate the expression of AR. In particular, YAP activation results in higher levels of c-Myc, which in turn up-regulates transcription of AR. YAP can also interact with the AR in the nucleus and facilitate its transcriptional activity [66], further supporting a role for aberrant signalling in PC. Furthermore, the down-regulation of YAP activity by treatment with IKBKE inhibitors also reduced proliferation, colony forming ability and AR activity in enzalutamide-resistant cell line models [23], therefore supporting the reactivation of the Hippo pathway as a method of targeting CRPC.

## 10. Conclusions and Perspectives

**Figure 3:**
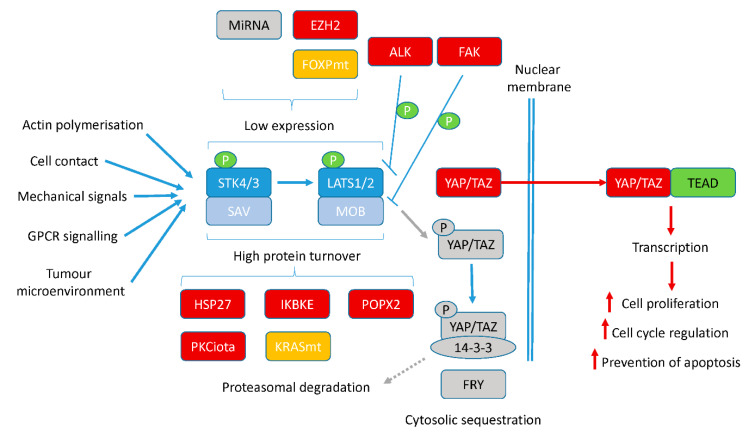
Potential therapeutic targets to reactivate the Hippo pathway. Modulation of upstream kinases STK3/4 and LATS1/2 occurs via post-translational modifications and transcriptional regulation. In cancer, a number of these molecules responsible for this regulation are altered. Molecules can be mutated (orange), overexpressed (red) or lost (grey) to result in high turnover or transcriptional silencing. This turns off the Hippo signalling cascade, allowing effector proteins, YAP/TAZ, to enter the nucleus and activate gene transcription, resulting in higher cell proliferation, cell cycle modulation, expression of other oncogenes such as c-Myc and AR and prevention of apoptosis, to name but a few processes.

The role of each component of the Hippo signalling pathway in PC development and progression is being uncovered. The importance of crosstalk between this pathway and well-established driver signalling pathways in PC is clear. Effects on AR, cancer stem cell biology, radioresistance, hypoxia-mediated signalling and response to the microenvironment all suggest that targeting this pathway therapeutically is warranted. With multiple new regulator molecules of this pathway being identified (Figure 3), a number of new therapeutic targets to restart Hippo signalling and turn off the activities of its effector proteins, YAP/TAZ, have been identified.

### 10.1. Therapeutic Options

The ability to target a dysregulated Hippo pathway therapeutically is growing ever closer. As reviewed in 2018 [120], a number of therapies can already down-regulate the activity of YAP/TAZ (Table 1). We can now add other therapeutic strategies to this list. These molecular targets include ALK, FAK, HSP27, IKBKE and POPX2. ALK inhibitors crizotinib and alectinib have both been described to be effective in the treatment of small cell carcinoma of the prostate where ALK activity is up-regulated [54]. FAK inhibition has recently been shown to abolish YAP activation in PC [121]. Indeed, a number of FAK inhibitors have been tested in clinical trials with promising results in prostate and other cancers [122,123,124]. The HSP27 antisense oligonucleotide, apatorsen (OGX427), has been used in Phase II clinical trials in patients with metastatic CRPC and demonstrated increased response rates compared to controls [125]. These studies suggest that targeting in humans is possible and that the effects of these therapies will provide patient benefit.

Verteprofin, which is approved by the FDA, is the most widely used YAP inhibitor, although its mode of action appears to be multimodal depending on the cellular background. Cell lines which do not express YAP still show a reduction in proliferation, which in one study was shown to be a consequence of impacting STAT3 activity [126]. However, results are promising nonetheless, but development of more specific YAP inhibitors is required. Similar findings are present for IKBKE inhibitors. Whilst the inhibitor Amlexanox has been successfully used in patients without major toxicities for other conditions, its specificity could be improved, but this may be challenging.

### 10.2. Outlook

With the field of Hippo signalling progressing so quickly, the prospect of seeing a therapeutic in a clinical trial for PC is real. A point for consideration is if patients have multiple defects in their Hippo pathway, for example, a loss of LATS1/2 expression and up-regulation of a YAP/TAZ-stabilising kinase, it may prove more effective for combinations of therapies to be administered in a personalised medicine approach. Therefore, how patients are stratified to receive such a treatment will be important based on the significance of post-translational modifications in the regulation of these pathways rather than transcriptional or mutational processes. Whilst there are still some questions remaining regarding the mechanistics and crosstalk of this pathway that are relevant to PC, the evidence for targeting this pathway therapeutically grows ever stronger.

## Figures and Tables

**Table 1:** Therapies which down-regulate YAP/TAZ activity.

Drug	Target	Mechanism
Verteporfin	YAP	Up-regulates 14-3-3
Dasatinib	Tyr inhibitor	Promotes proteasomal degradation of YAP/TAZ
Pazpanib	Tyr inhibitor	Promotes proteasomal degradation of YAP/TAZ
Cerivastin	HMGCoA reductase	Cytoplasmic sequestration of YAP/TAZ
XAV939	Tankynase	Decreased YAP/TAZ-mediated expression
Statins	HMGCoA reductase	Cytoplasmic sequestration of YAP/TAZ
C19		Activates MST1 and LATS1
MF-438	SCD1	

## Data Availability

Data analysed in this study are openly in CBioportal [14], Oncomine [46,69,70,71,14] and Protein Atlas [15].
